# Fatal crashes and rare events logistic regression: an exploratory empirical study

**DOI:** 10.3389/fpubh.2023.1294338

**Published:** 2024-01-05

**Authors:** Yuxie Xiao, Lulu Lin, Hanchu Zhou, Qian Tan, Junjie Wang, Yi Yang, Zhongzhi Xu

**Affiliations:** ^1^School of Public Health, Sun Yat-sen University, Guangzhou, China; ^2^Engineering Consulting Department, Changsha Planning and Design Institute Co., Ltd., Changsha, China; ^3^School of Traffic and Transportation Engineering, Central South University, Changsha, China; ^4^Institute of Transportation System Science and Engineering, Beijing Jiaotong University, Beijing, China; ^5^School of Transportation and Logistics, Southwest Jiaotong University, Chengdu, China; ^6^National Engineering Laboratory of Integrated Transportation Big Data Application Technology, Chengdu, China

**Keywords:** traffic safety, fatal crashes, rare events, logit model, binary classification

## Abstract

**Objective:**

Fatal road accidents are statistically rare, posing challenges for accurate estimation through the classic logit model (LM). This study seeks to validate the efficacy of a rare events logistic model (RELM) in enhancing the precision of fatal crash estimations.

**Methods:**

Both LM and RELM were employed to examine the relationship between pertinent risk factors and the incidence of fatal crashes. Crash-injury datasets sourced from Hillsborough County, Florida served as the empirical basis for evaluating the performance metrics of both LM and RELM.

**Results:**

The analysis revealed that RELM yielded more accurate predictions of fatal crashes compared to LM. Receiver operating characteristic (ROC) curves were constructed, and the area under the curve (AUC) for each model was computed to offer a comparative performance assessment. The empirical evidence notably favored RELM over LM as substantiated by superior AUC values.

**Conclusion:**

The study offers empirical validation that RELM is demonstrably more proficient in predicting fatal crashes than the LM, thereby recommending its application for nuanced traffic safety analytics.

## 1 Introduction

The persistently high mortality rates from traffic crashes have intensified their classification as a significant global public health issue (1, 2). According to the World Health Organization (3), fatalities attributed to traffic crashes witnessed a 25% increase, rising from 1.08 million in 1990 to 1.35 million in 2016. This uptick not only represents a societal tragedy but also imposes considerable economic strain on communities and families.

Numerous studies have been undertaken to explore the relationships between various risk factors—such as sex, age, educational attainment, weather conditions, and alcohol consumption—and the outcomes of traffic crashes (4–10). Given that crash severity is generally categorized by levels, discrete outcome models have been instrumental in investigating the correlations between fatal crashes and contributory factors (11–16).

Among the models utilized, the binary logit model (LM) is predominant. However, this approach has limitations when dealing with rare events, such as fatal crashes. For instance, the Hong Kong Transport Department's statistics from 2015 reveal that, of 16,170 injury-related crashes, only 117 were fatal, representing a meager 0.72% of the total dataset (17). Extant literature corroborates that LM tends to significantly underestimate the occurrence of such rare events (18).

Against this empirical backdrop, the present study deploys a rare events logistic model (RELM) to enhance the precision of fatal crash estimations. The RELM has been successfully applied in other domains such as geomorphology, social science, and epidemiology (19–21). To the authors' best knowledge, this study involves the inaugural application of RELM in the specific field of fatal crash estimation.

## 2 Methodology

### 2.1 Logit model

Logistic regression is the most used method in crash injury severity analyses. To model the relationship between fatal crashes and the risk factors, the outcome variable *y*_*i*_ in the *ith* crash was set to be one of the two values: *y*_*i*_ = 1 representing fatal crashes and *y*_*i*_ = 0 representing non-fatal crashes. The probability of *y*_*i*_ = 1 is denoted by Pr(*y*_*i*_ = 1), which is calculated using the following equation:

Logistic regression is the predominant method employed in the analyses of crash injury severities. To elucidate the relationship between fatal crashes and associated risk factors, we define the outcome variable *y*_*i*_ for the *i*th crash as binary: *y*_*i*_ = 1 signifies a fatal crash, while *y*_*i*_ = 0 indicates a non-fatal crash. The probability that *y*_*i*_ = 1, denoted as Pr(*y*_*i*_ = 1), is calculated using the logistic function:


(1) $$\mathrm{Pr}\left(y_{i}=1\right)=\;\frac{1}{1+e^{-\beta x_{i}^{'}}}$$

In Equation (1), $e^{-\beta x_{i}^{'}}$ encapsulates the linear combination of predictor variables, known as the utility function, which is expressed as:


(2) $$\beta x_{i}^{'}=\beta_{0}+\beta_{1}x_{1i}+\cdots +\beta_{k}x_{ki}$$

Here, *x*_*ki*_ represents the value of the *k*th variable for the *i*th observation and β_*k*_ is the corresponding coefficient.

There is another way to formulate the aforementioned question. Let us assume an unobserved continuous variable $y_{i}^{*}$, which represents the propensity of where a fatal crash occurred. $y_{i}^{*}$ follows a logistic distribution, which is close to normal (mathematically, the difference exists but is trivial). If we want to know the effects of *x*_*i*_, the standard approach is to run a regression with *x*_*i*_ as the dependent variable. To determine whether the crash is fatal or not, we observed whether this propensity is greater than a specific threshold. As documented by King and Zeng (19), this mechanism turns out to be the chief troublemaker in bias induced by rare events. The coefficients of β are estimated using the maximum-likelihood method with the following equation over a dataset of *n* observations:


(3) $$\mathrm{Pr}\left(y_{i}=1\right)=\;\frac{1}{1+e^{-\beta x_{i}^{'}}}$$

In Equation (3), $e^{-\beta x_{i}^{'}}$ is the multiple linear combinations of explanatory variables, which are also known as the utility function, and can be represented as:


(4) $$\beta x_{i}^{'}=\beta_{0}+\beta_{1}x_{1i}+\cdots +\beta_{k}x_{ki}$$

where *x*_*ki*_ denotes the value of variable *k* for sample *i* and β_*k*_ is the coefficient of variable *k*.

Alternatively, one may conceptualize the problem using a latent variable $y_{i}^{*}$, which signifies the propensity for a crash to be fatal. This latent variable follows a logistic distribution, which, despite its mathematical distinctiveness, is practically akin to a normal distribution. The impact of the predictors *x*_*i*_ is typically assessed by regressing them against this unobserved variable. The determination of the crash outcome—fatal or otherwise—is contingent upon whether the propensity surpasses a specified threshold. As highlighted by King and Zeng (19), this threshold mechanism introduces a primary source of bias in the presence of rare events. The logistic regression coefficients β are estimated by employing the maximum-likelihood estimation method applied across a dataset comprising *n* observations:


(5) $$L\left(\beta \right)=\prod_{i=1}^{n}\left[{\left(\frac{1}{1+e^{-\beta x_{i}^{'}}}\right)}^{y_{i}}{\left(1-\;\frac{1}{1+e^{-\beta x_{i}^{'}}}\right)}^{1-y_{i}}\right]$$

It is imperative to acknowledge that, in the analysis of rare events data, additional occurrences of the event of interest (coded as “1”) provide greater informational value than non-occurrences (coded as “0”). During the estimation phase, the standard error of the estimated coefficient β is derived from the variance:


(6) $$V\left(\hat{\beta }\right)=\frac{1}{\sum_{i=1}^{n}\pi_{i}(1-\;\pi_{i})x_{i}^{2}}$$

In Equation (6), the summation $\overset{n}{\underset{i=1}{\sum }}\pi_{i}\left(1-\pi_{i}\right)$ is notably influenced by the rarity of the event under study. The term π_*i*_(1−π_*i*_) attains its maximum when π_*i*_ = 0.5 and approaches zero as π_*i*_ converges to either extremity of the probability spectrum. Given that rare events data typically yield minuscule estimates of π_*i*_ for all observations, it is crucial to consider that these estimates will be substantially smaller than 0.5. Nonetheless, if the logit model possesses explanatory significance, the estimated probabilities π_*i*_ corresponding to the occurrences of “1” will be markedly higher than those associated with “0”. These estimates will also lie nearer to the apex of informational value at 0.5. Consequently, this results in the additional occurrences of “1” being more informative for the model than the additional occurrences of “0”.

### 2.2 Rare events logistic model

To ameliorate the bias in estimation attributed to the use of LM in rare events data, King and Zeng (18) introduced the RELM. RELM not only mitigates underestimation bias but also enhances the efficiency of data collection and reduces the requirements for data storage space during the sample selection phase.

#### 2.2.1 Sample selection

As highlighted in the preceding discussion, the LM exhibits suboptimal performance when instances of *y*_*i*_ = 1 are infrequent within the dataset. To address this limitation, a strategic alteration in data collection is proposed. By archiving all observations where a fatal crash occurred (*y*_*i*_ = 1) and a random subset of non-fatal crash observations (*y*_*i*_ = 0), we can refine the accuracy of the standard logit model's estimations.

#### 2.2.2 Adjustment of estimates for selection bias

To correct for selection bias inherent in choice-based sampling, two primary methods are employed: the prior correction and the weighting correction. The subsequent sections will elucidate these approaches in detail.

Research by King and Zeng (18) demonstrates that the logit model coefficients remain statistically consistent between population estimates and those derived from selected data. The objective of the prior correction method is to adjust the intercept $\hat{\beta_{0}}$ in the logit model using the following formula:


(7) $$\beta_{0}=\hat{\beta_{0}}-ln\left[\left(\frac{1-\tau }{\tau })(\frac{y}{1-y}\right)\right]$$

where τ represents the proportion of *y*_*i*_ = 1 within the population, while *y* signifies the proportion of *y*_*i*_ = 1 within the sampled dataset. The calculation of the probability of rare events occurrence is contingent upon accurate estimations of both β_0_ and β_*k*_, as indicated in Equation (1).

It is essential to note that the prior correction method necessitates the knowledge of τ, the population proportion of *y*_*i*_ = 1. In the context of this study, τ can be directly ascertained from the initial dataset of crash data. A principal benefit of the prior correction method lies in its user-friendliness; it can be readily implemented with any statistical software capable of fitting standard logistic models. For instance, the study by Ren et al. (22) leveraged this method to adjust estimates concerning the influence of various factors on red-light running behavior. Next, we will delineate an alternative approach that can augment the efficacy of the logistic model (LM) when used in conjunction with prior correction.

The weighting correction involves assigning weights to the data to balance the discrepancies in the proportions of *y*_*i*_ = 1 between the sample and the population, which arise from choice-based sampling. This method entails optimizing a weighted log-likelihood function rather than the conventional log-likelihood function:


(8) $$\begin{array}{l} \ln L_{w}\left(\beta |y\right)=\omega_{1}\sum_{\left\{y_{i}=1\right\}}\ln\pi_{i}+\omega_{0}\sum_{\left\{y_{i}=0\right\}}\ln(1-\pi )=\\ \;-\sum_{i=1}^{n}\omega_{i}\ln\left(1+e^{(1-2y_{i})x_{i}\beta }\right) \end{array}$$

In this context, the weights ω_1_ and ω_0_ are defined as $\omega_{1}=\tau /y$ and $\omega_{1}=\tau /y$, respectively, where ω_*i*_ = ω_1_*y*_*i*_+ω_0_(1−*y*_*i*_). The parameters τ and *y* retain their definitions from the “prior correction” section.

Although this method may appear more complex than the prior correction technique, Equation 6 is formulated to enable researchers to apply it using any standard logit software package.

Xie and Manski (23) posited that weighting correction could surpass prior correction in effectiveness when the available sample is substantial, and there is a mis-specification of the functional form. Conversely, Amemiya and Vuong (24) indicated that, while weighting correction may be marginally less efficient than prior correction, the difference in efficiency is typically negligible.

#### 2.2.3 Computing probability estimates

Subsequent to implementing the prior correction and weighting methods, we adapt modifications suitable for both cohort and choice-based sampling designs in rare events logistic models. The bias in the estimated coefficients $\hat{\beta }$ is appraised using the weighted least-squares method, formulated as:


(9) $$bias\left(\hat{\beta }\right)=\;{\left(X^{'}WX\right)}^{-1}X^{'}W\xi$$

where $\xi_{i}=0.5Q_{ii}\left(\left(1+\omega_{1}\right)\hat{\pi_{i}}-\omega_{1}\right)$ symbolizes an adjustment factor, where *Q*_*ii*_ are the diagonal constituents of the matrix *Q* = *X*(*X*′*WX*)−1*X*′ and $W=diag\left\{\hat{\pi_{i}}\left(1-\hat{\pi_{i}}\right)\omega_{i}\right\}$ is a diagonal matrix with elements $\hat{\pi_{i}}\left(1-\hat{\pi_{i}}\right)\omega_{i}$. Consequently, the adjusted coefficients $\tilde{\beta }$ are calculated as follows:


(10) $$\begin{array}{l} \hat{\beta }-bias\left(\hat{\beta }\right)=\tilde{\beta } \end{array}$$

The final corrected probability *P*_*i*_ can be approximated by the following expression:


(11) $$\begin{array}{l} P_{i}={\tilde{\pi }}_{i}+C_{i} \end{array}$$

where the correction term *C*_*i*_ is delineated as follows:


(12) $$C_{i}=\left(0.5-{\tilde{\pi }}_{i}\right){\tilde{\pi }}_{i}\left(1-{\tilde{\pi }}_{i}\right)X_{i}V\text{​}\left(\tilde{\beta }\right)X_{i}^{'}$$

Within this equation, $V\left(\tilde{\beta }\right)$ denotes the estimated variance-covariance matrix of the adjusted coefficients $\tilde{\beta }$. *X*_*i*_ = (1, *x*_*i*_) represents the vector of predictors, including the intercept for the *i*th observation, and $X_{i}^{'}$ is its transpose. Collectively, these amendments constitute the methodology of the RELM. To the authors' knowledge, this is the first instance of applying RELM within the domain of fatal crash estimation.

## 3 Data description

Data on crash-related injuries that occurred in the year 2006 in Florida were procured from the Florida Department of Highway Safety and Motor Vehicles (DHSMV). The dataset encompasses 107,464 driver-vehicle units implicated in 53,732 traffic incidents. A meager 0.34% of these incidents resulted in fatalities, highlighting their infrequency. The variables under scrutiny encompass critical attributes, such as those associated with the driver, the vehicle, the roadway, and the environmental context, as delineated in prior research (25–28). Table 1 delineates the variables and their corresponding characteristics as encapsulated within the Florida dataset.

**Table 1:** Variables contained in the dataset.

**Factor**	**Attributes**	**Count**	**Proportion**
Injury severity	Fatality	363	0.34%
	Non-fatal or no injury	107,101	99.66%
Driver age	Under 25 years	27,685	25.76%
	25–65 years	69,677	64.84%
	Above 65 years	10,102	9.40%
Driver sex	Male	60,567	56.36%
	Female	46,897	43.64%
Alcohol/drug use	No drink or drugs	103,218	96.05%
	Drink or drugs	4,247	3.95%
Seat belt	Not using a seat belt	5,531	5.15%
	Using a seat belt	101,933	94.85%
Driver fault	At fault	44,690	41.59%
	Not at fault	62,774	58.41%
Vehicle year	1996–2006	81,704	76.03%
	< 1996	25,760	23.97%
Vehicle type	Passenger car	73,492	68.39%
	Van	8,550	7.96%
	Light truck/pick-up	21,832	20.32%
	Medium/heavy truck	3,590	3.34%
Speed ratio	< 0.5	36,676	34.13%
	0.5–1.0	65,641	61.08%
	>1	5,147	4.79%
POI	Level 1	74,949	69.74%
	Level 2	18,381	17.10%
	Level 3	13,816	12.86%
	Level 4	318	0.30%
Day of week	Weekday	81,930	76.24%
	Weekend	25,534	23.76%
Location	Rural	50,384	46.88%
	Urban	57,080	53.12%
Light condition	Daylight	81,980	76.20%
	Dark	25,484	23.80%
Weather	Clear	79,720	74.18%
	Not clear	27,744	25.82%
Surface	Dry	94,726	88.15%
	Not dry	12,738	11.85%
Vision	Not obscured	99,718	92.79%
	Obscured	7,746	7.21%
Highway	Divided highway	60,014	55.85%
	Undivided highway	47,450	44.15%

Number of observations = 107,464.

**Figure 1:**
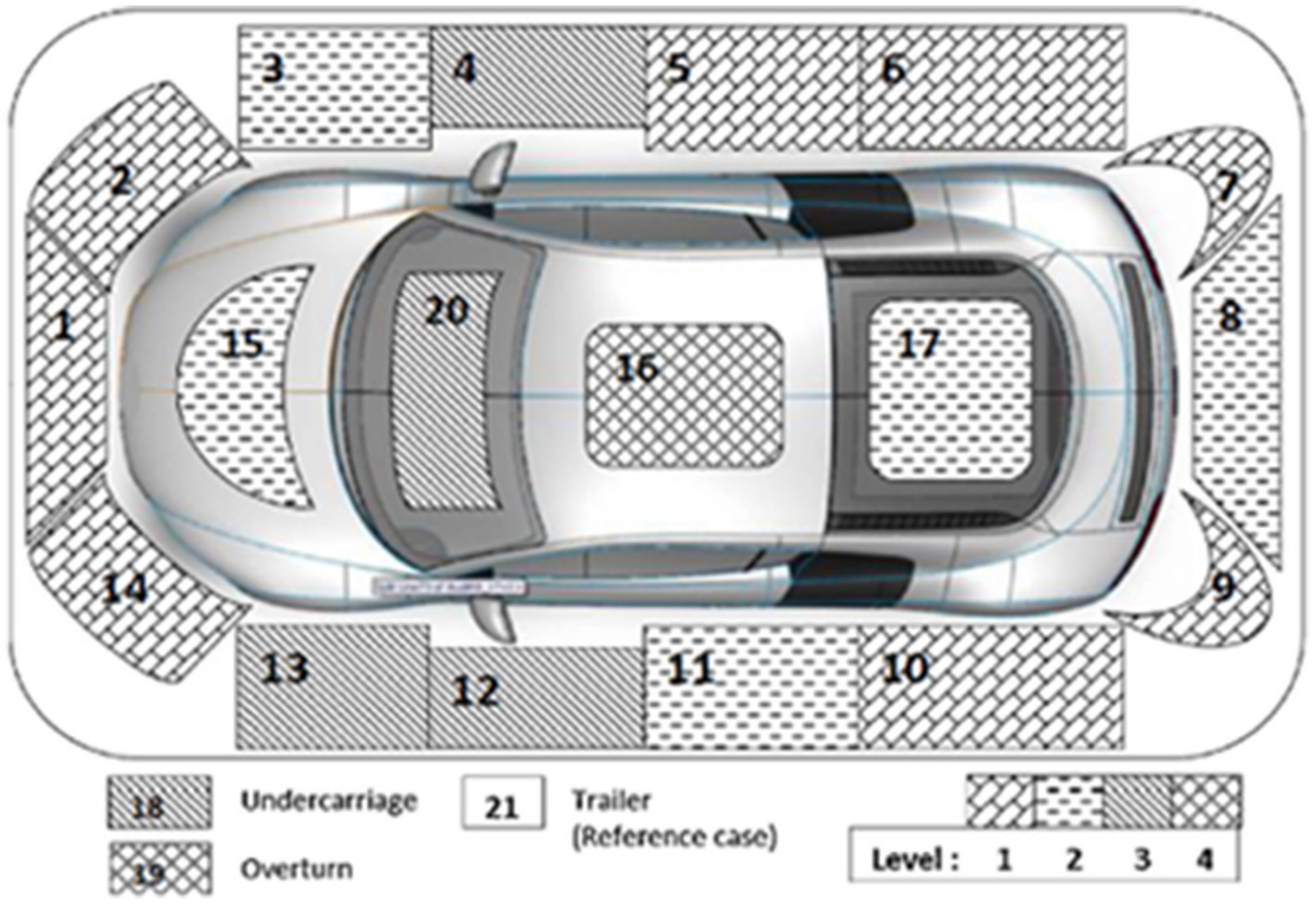
An illustration of the points of impact.

Notably, the “speed ratio”—defined as the quotient of the estimated speed prior to the collision and the statutory speed limit post-collision—is posited to correlate positively with injury severity (25). Furthermore, the analysis includes “points of impact” (POIs) on the vehicle, enumerated in the Florida crash reports and illustrated in Figure 1. These POIs are categorized in alignment with the schema proposed by Huang et al. (29), where Level 1 encompasses nine POIs (nos. 1–2, 5–7, 9–10, 14, and 21) located peripherally relative to the driver's seat, such as the front and rear passenger sides. Level 2 consists of five POIs (nos. 3, 8, 11, 15, and 17) situated in closer proximity to the driver than those in Level 1. Level 3 includes POIs (nos. 4, 12–13, 18, and 20), which are nearest to the driver, comprising the windshield and the front passenger and driver sides. The final category, Level 4, is assigned to two POIs (nos. 16 and 19).

## 4 Model evaluation

In the evaluation of our models, namely, RELM and the LM, we quantify the predictive performance using the area under the receiver operating characteristic curve (AUC-ROC). The AUC is a widely accepted metric for model performance evaluation, particularly in binary classification problems. It provides an aggregate measure of performance across all possible classification thresholds. The calculation of the AUC involves plotting the true positive rate (sensitivity) against the false positive rate (1-specificity) at various threshold settings (30). The AUC value ranges from 0 to 1, where an AUC of 1 indicates perfect predictive accuracy and an AUC of 0.5 suggests performance no better than random chance.

To estimate the AUC accurately, we employ the trapezoidal rule for numerical integration as this method is well-suited for the discrete data points that characterize an empirical ROC curve (31). Furthermore, we validate the robustness of our AUC estimates through K-fold cross-validation, which mitigates the potential for overfitting by ensuring that each observation is used for both training and validation. This process involves partitioning the data into *K* equal-sized segments, training the model on *K*−1 segments, and validating it on the remaining segment. This is repeated *K* times, with each segment used exactly once for validation. The average AUC across all *K* iterations provides a reliable estimate of the predictive performance of the models. In this study, *K* was set to 5.

## 5 Results

### 5.1 Data sampling

As previously mentioned, the initial step involves the partial extraction of the complete dataset for regression analysis. This entails retaining all instances of fatal crashes while selectively including a subset of non-fatal crashes. To ascertain the optimal proportion of “1” events in the newly constituted dataset, this study computes the coefficients employing both the prior correction and weighting correction methods, incrementally adjusting by 1% within a range from 0.05 to 0.95. The variation in classification accuracy is further assessed using two metrics: the accurate classification rate (ACR), defined as the quotient of correctly identified fatal accidents to the total number of actual fatal accidents; and the false classification rate (FCR), computed as the quotient of erroneously classified incidents to the total number of events.

**Figure 2:**
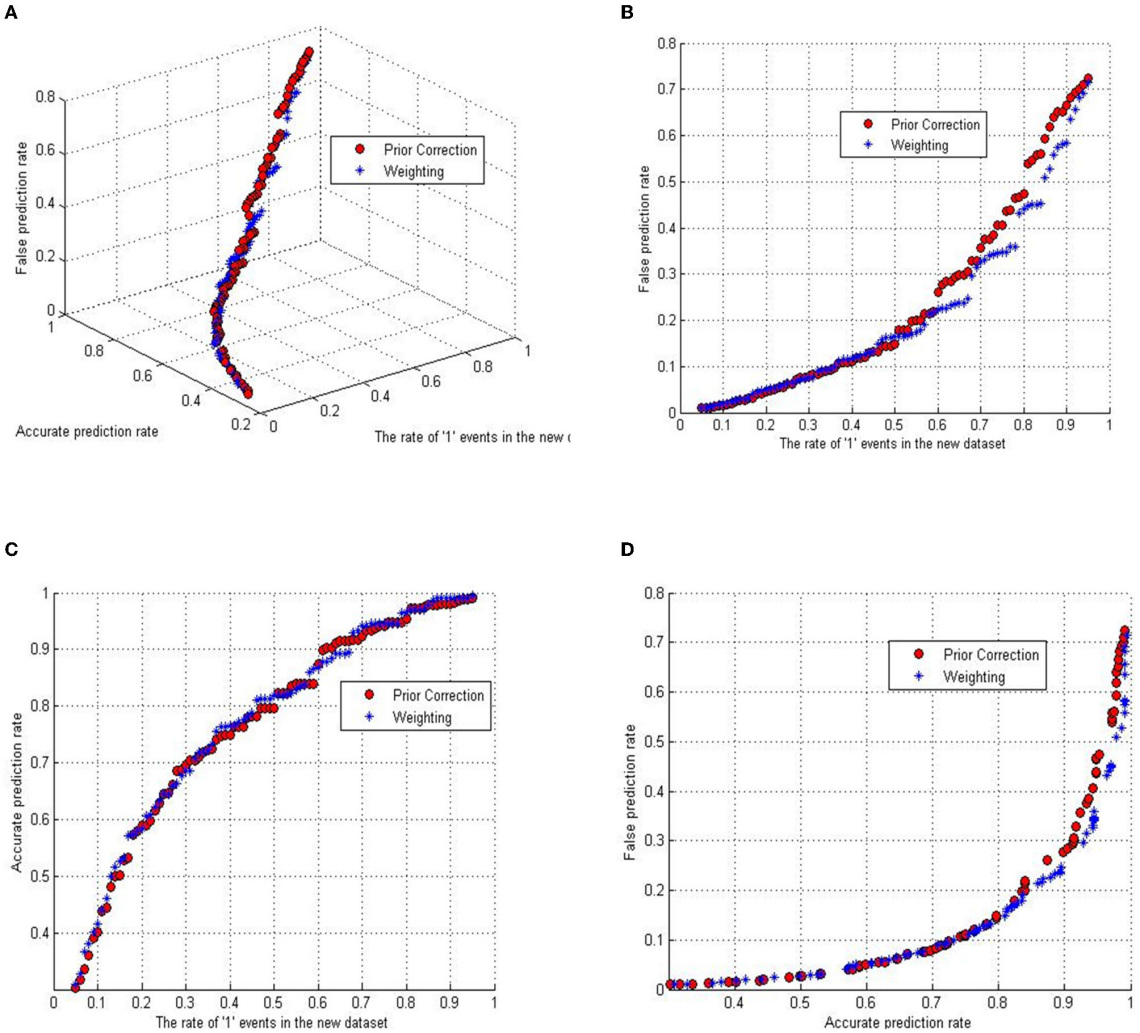
The relationship between measurements and the ratio of rare events. **(A)** The relationship between accurate classified rate, false classified rate, and the fatal event ratio. **(B)** The relationship between false classified rate and the fatal event ratio. **(C)** The relationship of accurate classified rate versus the fatal event ratio. **(D)** The relationship between accurate classified rate and false classified rate.

Figure 2 delineates the interplay between the three aforementioned variables: ACR, FCR, and the ascending fraction of “1” events in the sampled data. The depiction includes red dots representing outcomes via the prior correction method and blue stars indicating results from the weighting method. A 3D subgraph within Figure 2A visualizes the pairwise interactions among these factors, with the remaining panels (Figures 2B–D) presenting projections along different axes.

Analysis of Figure 2 reveals a close alignment between the trajectories of ACR and FCR across both correction methodologies. A trend emerges where an elevated ACR correlates with a heightened FCR. Notably, the ACR ascends more precipitously than the FCR within the “1” event ratio spectrum from 0.05 to 0.5, while this growth rate inverts for ratios between 0.5 and 0.95.

**Figure 3:**
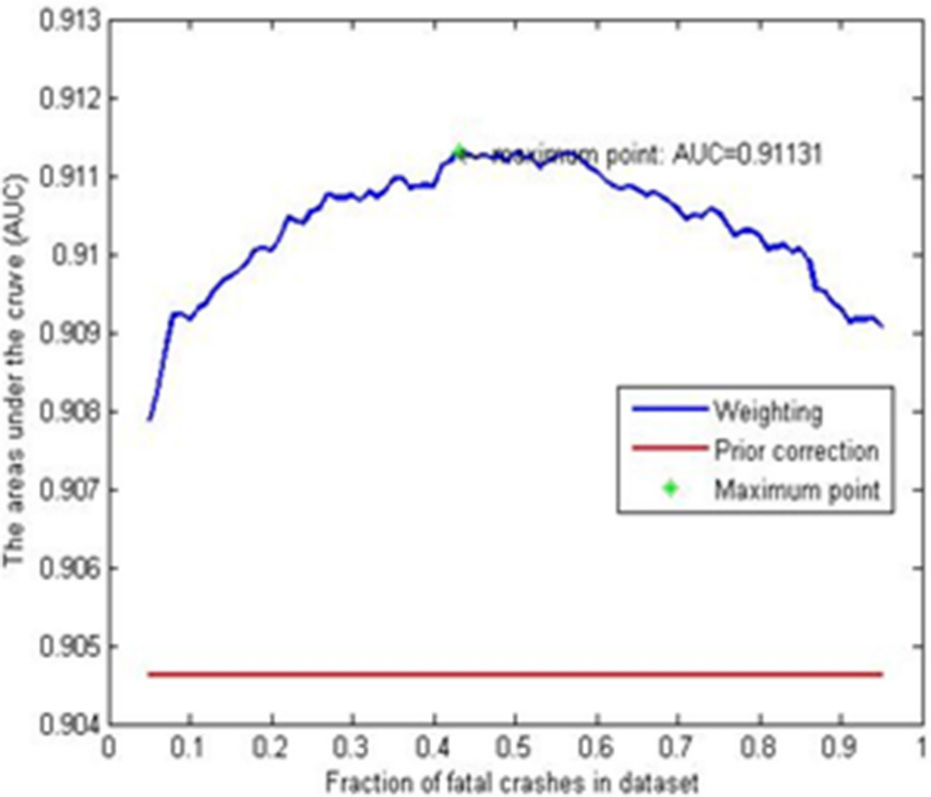
AUC values for the weighting method and the prior correction method.

Figure 3 presents the AUC for both methods across varying proportions of fatal to non-fatal crashes. The diagram indicates that the AUC for the prior correction method remains unaffected by the percentage of “1” event post-selection. In contrast, the weighting method demonstrates superior predictive performance at most “1” event ratios. Green stars mark the coordinates with the maximum AUC values, which inform the selection of rates for the weighting method in the rare events logistic model—specifically, 43% in the corrected dataset. For the implementation of the rare events logistic model, the Stata statistical software package was employed.

### 5.2 The parameters of models

The parameter estimates for the RELM and the LM are consolidated in Tables 2, 3, respectively. These tables encapsulate the significant parameters deduced from the empirical analysis, illustrating that the magnitude and direction of the coefficients for both models are largely consistent. The significance and impact of the variables, with the salient exception of the POI, are in concordance with the injury severities reported in antecedent research, notably by Zeng and Huang (26).

**Table 2:** Model parameters of RELM.

**Variable**	**Coefficient**	**95% confidence interval**	
Age level 3 (above 65 years)	2.553	2.192	2.914
Drug use	1.854	1.495	2.213
Using seat belt	2.279	1.940	2.619
Veh_year (< 2006)	−0.415	−0.720	−0.110
Medium/heavy truck	0.492	0.160	0.824
Speed ratio (< 0.5)	−1.703	−2.226	−1.180
Speed ratio (0.5–1.0)	−1.308	−1.791	−0.825
POI	1.536	1.217	1.855
Not at fault	−2.609	−3.118	−2.100
Rural	1.017	0.709	1.325
Daylight	−0.843	−1.160	−0.526
Constant	0.036	−0.615	0.687

**Table 3:** Model parameters of LM.

**Variable**	**Coefficient**	**95% confidence interval**	
Age level 3 (above 65)	1.970	1.722	2.219
Drug use	2.016	1.711	2.322
Using seat belt	2.122	1.873	2.371
Veh_year (< 2006)	−0.281	−0.512	−0.050
Medium/heavy truck	0.411	0.158	0.663
Speed ratio (< 0.5)	−0.926	−1.294	−0.557
Speed ratio (0.5–1.0)	−0.984	−1.315	−0.653
POI level 3	1.489	1.260	1.717
Not at fault	−2.957	−3.397	−2.516
Rural	1.003	0.773	1.233
Daylight	−0.396	−0.639	−0.154
Constant	−5.934	−6.418	−5.451

Our analysis of driver demographics indicates a heightened risk of fatality for older drivers following a collision, corroborating the findings from existing literature that underscores age as a critical determinant in traffic injury severity. In relation to vehicular and environmental factors, the data suggest that more recent vehicle models correlate with a reduction in injury severity, supporting the premise that advancements in vehicular safety technologies have ameliorated crash outcomes. In clear contrast, while operators of medium/heavy trucks exhibit a lower fatality likelihood, drivers of passenger cars show an increased fatal outcome propensity. This disparity may be attributable to inherent variations in vehicle safety features, structural mass, and design specifications.

### 5.3 Comparative analysis of classification efficacy

Table 4 delineates the predicted outcomes derived from both the RELM and the LM, incorporating statistically significant variables at the 0.05 level into the classification procedure. The predictive classifications of the models are juxtaposed against the actual incident outcomes, with Table 4 providing a comprehensive summary of these predictions. The data articulated in Table 4 highlights the superior performance of RELM in comparison with LM. A notable deficiency of LM is its significant underestimation of fatal accident risk, failing to identify any incident as fatal. In contrast, RELM achieves an accurate classification rate of 77.7%. Despite an increase in the false alarm rate by 12.8%, RELM is deemed tolerable when juxtaposed against the grave implications of underestimating fatal accidents; for instance, Aguero-Valverde (32) equates the impact of 1 fatal crash to that of 20 property-damage-only (PDO) crashes.

**Table 4:** The prediction results of LM and RELM.

	**LM**	**RELM**
Number of crashes	107,464	
Number of fatal crashes	363	
Number of predicted fatal crashes	0	14,013
Number of true positives	0	282
Accuracy	/	77.7%

**Figure 4:**
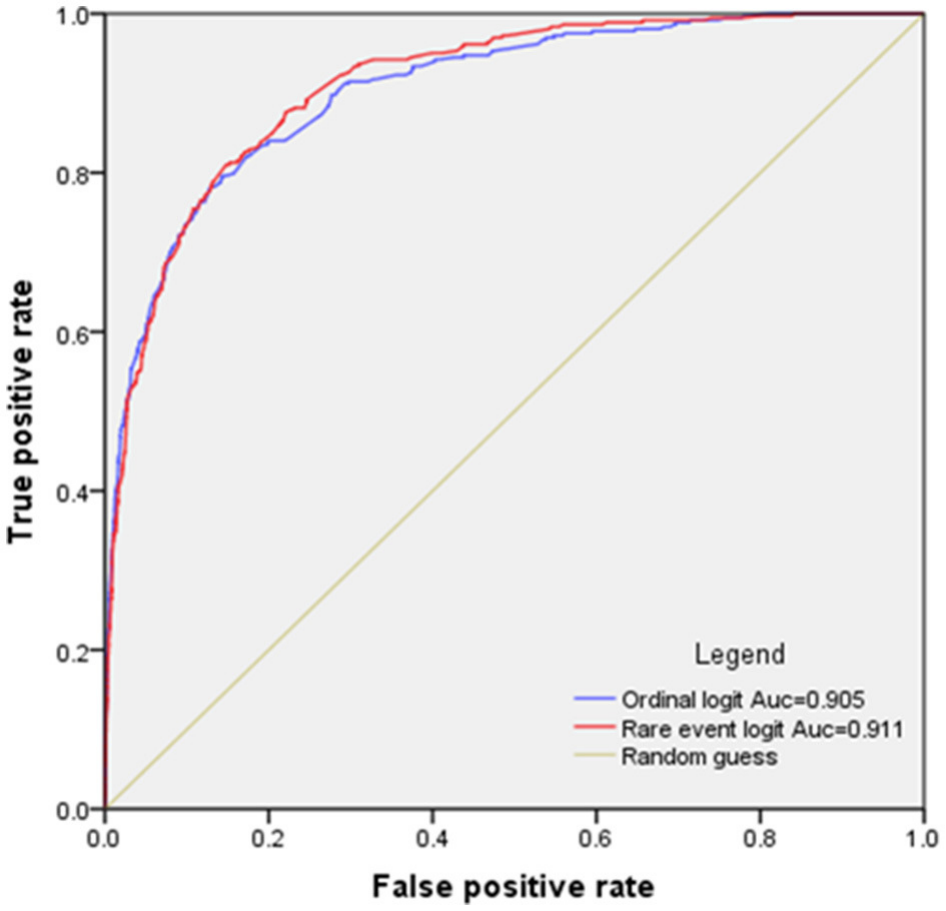
ROC curves for RELM and LM methods.

An extended evaluation of the performance of the two models was conducted through the ROC curves, as exhibited in Figure 4. The predictive accuracy for fatal and non-fatal cases is contingent upon a predetermined probability threshold. An observation is designated as a fatal accident if its predicted probability transcends this threshold; otherwise, it is categorized as non-fatal. The ROC curves graphically represent the tradeoff between the true positive rate and the false positive rate as the threshold varies from 0 to 1. The AUC for each model is computed, revealing that the ROC curve for the RELM generally resides above that of the LM for thresholds below 0.8, indicative of enhanced predictive accuracy of RELM. Moreover, a juxtaposition of the AUC values in Figure 4 confirms the integrated predictive superiority of the RELM model over the LM.

## 6 Discussion

This study employs the rare events logistic model to scrutinize the relationship between various risk factors and the incidence of fatal road accidents in Florida. The analysis identifies six variables—older adult casualties, substance abuse, non-usage of safety equipment, passenger car, POI at level 3, and rural accidents—as positively correlated with driver fatalities. Conversely, five variables—vehicle age, speed ratios 1 and 2, driver at fault, and daylight incidents—exhibited a negative correlation with accident risk.

The findings unequivocally show that RELM supersedes LM in estimating fatal crash risks. As hypothesized, LM systematically underestimates these risks, a shortfall that RELM substantially rectifies, achieving an accuracy rate of ~80%. While a slight increase in false classification is noted, this tradeoff is deemed acceptable given the enormity of losses associated with each fatal accident. The AUC values further corroborate the superior performance of RELM over LM in this context.

The findings of this study have several implications for stakeholders involved in road safety. It is recognized that annual inspections cannot alter the fundamental crashworthiness of older vehicles; however, ensuring that aging vehicles are maintained can help mitigate risks where possible. Nevertheless, the intrinsic limitations in safety offered by older vehicle designs compared to their modern counterparts must be acknowledged. Thus, stakeholders should focus on enhancing public awareness regarding the potentially increased risks associated with older vehicles and should advocate for policies that encourage the use of vehicles with advanced safety features. For demographic groups such as older adult drivers and men who are statistically at a greater risk, targeted safety campaigns and driving aids could be beneficial. This could involve educational initiatives that promote defensive driving techniques and raise awareness about the increased risk factors these demographics face. Furthermore, urban planners and transportation authorities should take into account the findings regarding speed limits. While not the sole factor, the data suggest that higher speed limits can contribute to the severity of crashes. Therefore, a holistic approach to road design that incorporates traffic calming measures and considers the impact of speed on traffic incident severity is warranted. These measures could help in reducing the likelihood of fatal outcomes in crashes.

This study is subject to certain constraints that warrant acknowledgment. The classification of POIs into predefined levels, a method predicated on established literature, may not capture the entirety of POIs that may significantly influence crash severity. The dataset utilized provided a finite array of POIs, thereby omitting potentially crucial impact points not recorded within it. This omission could lead to a partial portrayal of crash dynamics. Moreover, spatial correlation, a factor that could yield valuable insights into the patterns and causes of fatal crashes, was not incorporated into the RELM used in this analysis. Other influential variables, such as law enforcement strategies and traffic volume data, were also not included in our dataset. The absence of these variables limits the breadth of our analysis, potentially affecting the robustness of our findings. Acknowledging these limitations, future investigative efforts in this field should endeavor to integrate a more detailed classification of POIs, alongside variables capturing spatial correlation, law enforcement efforts, and traffic metrics. Such enhancements in data collection and model sophistication would provide a more holistic understanding of the factors contributing to fatal crash outcomes.

## Data availability statement

The data is not available to the public due to data privacy policy. Requests to access these datasets should be directed to ZX, xuzhzh26@mail.sysu.edu.cn.

## Author contributions

YX: Writing—original draft. LL: Writing—original draft. HZ: Writing—original draft. QT: Writing—review & editing. JW: Writing—original draft. YY: Writing—original draft. ZX: Writing—original draft, Conceptualization, Formal analysis.
